# Looking for Novel Natural Gels to Improve Cleaning Methods for Bronze Leachates on Marble

**DOI:** 10.3390/gels9110843

**Published:** 2023-10-25

**Authors:** Iñaki Vázquez-de la Fuente, Inés Barbier, Sara Puente-Muñoz, Nagore Prieto-Taboada, Gorka Arana, Juan Manuel Madariaga

**Affiliations:** Department of Analytical Chemistry, Faculty of Science and Technology, University of the Basque Country UPV/EHU, P.O. Box 644, 48080 Bilbao, Basque Country, Spain; i.barbierdeolano@gmail.com (I.B.); spuente004@ikasle.ehu.eus (S.P.-M.); nagore.prieto@ehu.eus (N.P.-T.); gorka.arana@ehu.eus (G.A.); juanmanuel.madariaga@ehu.eus (J.M.M.)

**Keywords:** agar, kudzu, konjac, chelating agent, citrate, EDTA, leachates, copper

## Abstract

Marble is one of the materials most susceptible to copper leaching, resulting in easily identifiable turquoise stains on the marble. This problem is particularly relevant when we are talking about marble structures of heritage value. For this reason, conservators look for cleaning materials that are specific to the structure to be treated without damaging the original surface. Materials such as agar have been studied for a long time. Agar creates a controlled water release system that adapts to the needs of conservators who seek the greatest possible cleanliness without damaging the material to be treated. To improve the cleaning, chelating agents such as EDTA are added to the agar composition. However, the microbiological growth and the damage it produces to the original material are disadvantages to take into account. In order to solve these problems, other natural materials with cleaning potential such as kudzu and konjac gels were studied in combination with other chelating agents such as citrate, oxalate, and gluconic acid. For the characterization and evaluation of copper cleaning, various analytical techniques were used, including Raman spectroscopy, colorimetry, X-ray fluorescence (XRF), and inductively coupled plasma mass spectrometry (ICP-MS). In this study, both konjac and kudzu emerged as promising alternatives to agar, revealing distinctive features such as simplified preparation methods and inherent antimicrobial properties. The EDTA chelator was found to be the most harmful for marble surfaces, as it extracted a greater amount of calcium from the marble during application of the gels doped with it. Citrate and gluconic acid have been identified as a promising substitute to prepare doped gels for the removal of copper stains. These compounds exhibit comparable or potentially superior cleaning capabilities than EDTA, with no negative side effects.

## 1. Introduction

One of the significant challenges faced by conservators involves cleaning heritage objects. It is essential to preserve the original appearance of built heritage, which is why this task demands precision [1,2]. This cleaning must be carried out with care, always respecting the original structure of the work, to avoid any deterioration during its treatment. Copper stains are one of the biggest problems for conservators working to alleviate this problem. These stains, which can dye the surface of walls and sculptures, are usually caused by the mobilization of copper ions near bronze structures [3,4,5,6,7]. These ions eventually crystallise and form salts that generate a patina that is clearly distinguishable from the surface on which it crystallises due to its characteristic turquoise (copper) colours.

Porous materials such as marble are specially affected by copper patinas because it is quite common the use of this material as a support for bronze sculptures. The main challenge posed by copper leaching in heritage marble structures is the difficulty of removing it without damaging the marble in the process. For this reason, marble has been the target of chemical cleaning methods to effectively remove the formed copper salts [8]. In this sense, hydrogel-based methodologies are often used to create a controlled water release system to promote the cleaning of the surfaces [3,9,10]. Hydrogels can be composed of natural or synthetic compounds, and agar-based hydrogels have been widely used for cleaning stone materials [3,9,11,12]. In fact, these gels seem to have intrinsic characteristics that help clean the surfaces more than can be explained as cleaning containers [9]. Some hydrogels show the ability to clean without any addition of other compounds but there is the possibility of adding cleaning reagents that can improve their cleaning action. In the case of metallic staining, chelating agents have been used widely [13] and in particular ethylenediaminetetraacetic acid (EDTA) with very good results [3,9,10,12].

However, although agar gels have been extensively studied for their specific properties, they have disadvantages such as the need of heat to prepare them. This limits their use in the field, as a heat source would be required, and it can also be a problem if the gel is doped with a heat-sensitive compound. Another disadvantage is the possible presence of gel residues after applying the gel that could lead to the proliferation of microorganisms that can damage the restored work [9,14,15]. Regarding EDTA, it has been shown that it can form complexes with the calcium present in the structure of marble, making its use in this type of material potentially damaging, as the surface of the marble could be affected, despite its promising potential in restoration [12,16].

Taking all of these matters into account and following the principles of green chemistry, experimentation with increasingly ecological materials is being promoted for the sustainable restoration of cultural heritage [17]. In this sense, the IBeA research group has been investigating new alternatives such as natural hydrogels that can solve all or at least alleviate some of the problems mentioned. These alternatives include kudzu, which has biocidal potential [18,19]. It is extensively used in the cosmetics and food industries to produce semi-rigid gels in a similar way to agar. Being the most studied of the group, kudzu experiments have been carried out such as its application on paper with good results as an alternative to other commercial starches [18]. Our group is also studying the use of konjac, which is also traditionally used in food and cosmetics and can be used for the production of semi-rigid gels. It has already been used to absorb Cu(II) in waste waters in combination with other chemicals [20] and its use in restoration is beginning to be investigated [21]. Therefore, these natural materials can be effective alternatives to agar to improve the cleaning methodologies. Regarding the chelating agents, as previously mentioned, the most common is EDTA, but due to its disadvantages, it is usually preferable to use other reagents, such as citrate, for the cleaning of calcareous material [16].

For all these reasons, this study conducted a two-dimensional study comparing the cleaning potential of bronze leachates in marble between the new natural gels (kudzu and konjac) that were mixed, or not, with more environmentally friendly chelating agents and the reference cleaning methodology (agar with EDTA). The objective is to evaluate the potential of the target natural gels in combination with alternative chelators to EDTA by generating copper leachates in marble mock-ups. Spectroscopic analysis techniques were used to characterise the marble and patinas and to evaluate the efficacy.

## 2. Results and Discussion

### 2.1. Gels Preparation

During the preparation of the gels, several differences were observed between the gels. To ensure uniform consistency among the three types of gels, the proportions of konjac, kudzu, and agar were adjusted to 6%, 12%, and 2%, respectively. Consequently, agar facilitated a higher storage of water per unit area of gel. An increase in polymer concentration leads to a decrease in the capability of agar gels to release water at the gel-stone interface, which could have a negative impact on surface cleaning [3]. The agar composition percentage was determined from existing literature. Different compositions were tested for the other gels until a stiffness resembling that of agar was achieved. Reducing the concentration of the gel resulted in inadequate stability, resulting in excessively viscous and fluid gels. On the other hand, increasing concentrations reduced the gel’s adhesive properties. The gels exhibited different pH levels and the EDTA-doped gels were the most acidic while the others were around pH 7 (Table 1). Although there are differences in the pH without chelators, kudzu produces the most acidic gel; these differences disappear with the addition of reagents. In this sense, it was decided to avoid the use of buffers to simplify the composition of the gels.

The agar gels were easy to prepare obtaining rigid and smooth gels. On the downside, these gels were the most fragile and had the least adhesive power, which is something to bear in mind when applying to a vertical surface.

Kudzu was the most problematic to prepare. Firstly, during preparation, it was difficult to find the exact moment at which to remove it from the heat source, resulting in several failed attempts. If the gel remained on the heat for too long without frequent stirring, it would harden excessively and form lumps, leading to an uneven final gel consistency. This problem was resolved after a few attempts. As the ideal time to remove the kudzu and water mixture from heat approached, it began to turn whitish, making it easier to identify. Secondly, the gel’s viscosity caused some of it to be lost attached to the container in which it was prepared, making the preparation less reproducible. Nevertheless, it should be emphasised that after a few minutes, the gel reached optimal rigidity and had greater adhesion than the agar. Another important aspect is the antimicrobial capacity of this gel. This was verified by a simple test in which the three types of gel were left for 3 days and kudzu was the only one that showed no visible fungal colonies.

Konjac also presented the same problem related to viscosity in its preparation, although this gel presented a great advantage. Heating is unnecessary to prepare konjac gels; it is only required to mix it with water at room temperature. Considering that many pieces of copper-stained marble cannot be transported for restoration, this feature is very interesting as it allows the preparation of gels in situ, and it also makes it possible to adapt their composition to the problem to be treated by the restorer. Furthermore, its viscous nature is an advantage, allowing it to be applied to detailed surfaces where a rigid gel cannot be in contact with the entire surface. Additionally, the gels can be doped with temperature-sensitive compounds, which include many compounds with antimicrobial properties.

### 2.2. Marble Mock-Up Cleaning

#### 2.2.1. Mock-Up Characterization

**Figure 1:**
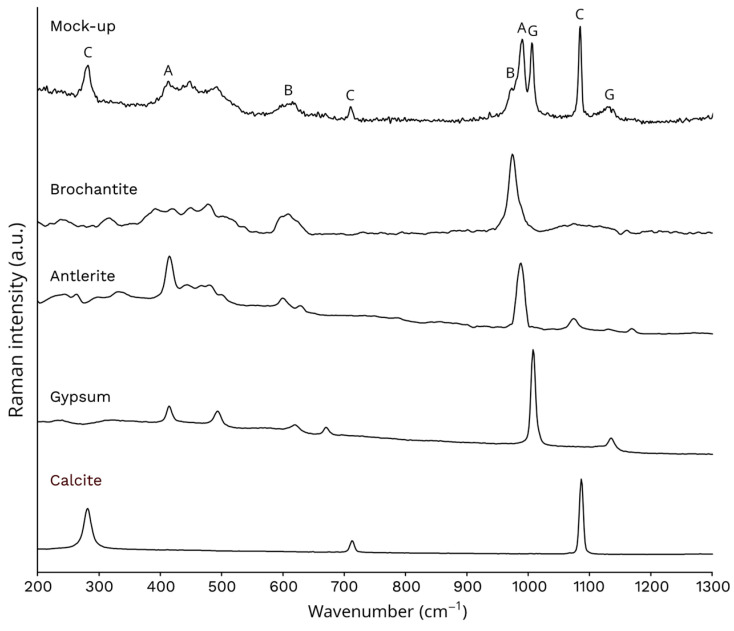
Raman spectrum obtained from a T1 stage mock-up and the standards of the compounds identified in it (B—brochantite; A—antlerite; G—gypsum; and C—calcite).

Raman spectra obtained from the original marble (T0) confirmed that they were calcite (CaCO_3_, 280, 712, and 1085 cm^−1^). On the other hand, the spectra obtained after immersion of the mock-ups in the CuSO_4_ solution (T1) revealed the presence of different copper (II) sulphate minerals. As shown in Figure 1, the spectra presented the main band of brochantite (Cu_4_SO_4_(OH)_6_, 975 cm^−1^) and the main and secondary bands of antlerite (Cu_3_(SO_4_)(OH)_4_, 988 and 415 cm^−1^). The main band of gypsum (CaSO_4_·2H_2_O, 1008 cm^−1^) was also observed, indicating that the marble had undergone degradation during the ageing process. The compounds found are typical of those found in real bronze leaching scenarios where copper is mobilised and deposited on the marble surface [3,7,22]. Therefore, taking into account these results, the chosen patina creation method was appropriated.

Another important aspect was the homogeneity of the created patina over the entire surface of the mock-ups. This was confirmed by colorimetry since at T1, the colour variation (ΔE) had a relative standard deviation (RSD) between all the mock-ups of 8.5%, which seems to be an acceptable dispersion for the creation of the patinas. Moreover, by using XRF, comparing the values obtained for Cu measured in all the mock-ups for the same area, the reproducibility of the Cu patina was better, with an RSD of 3%. This value is different from the colorimetry probably due to the different reproducibility of the techniques. This is because the colorimetry compared different small spots and in the case of the XRF images from all the studied areas were evaluated. In any case, the reproducibility of the mock-ups was very good.

#### 2.2.2. Cleaning Evaluation

**Figure 2:**
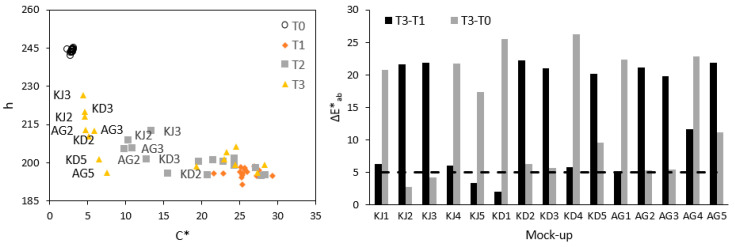
Chroma (C*) and hue (h) values of the mock-ups at each stage of the experiment (**left**). Graph of the colour variation (ΔE*) of the copper cleaning (**right**). It shows the change in colour after cleaning (T3-T1) and the similarity in colour between the original and cleaned mock-ups (T3-T0). ΔE* values greater than 5 (dotted line) indicate an easily perceptible change in colour.

The removal of copper from the surface of the mock-ups was measured and quantified by colorimetry and XRF. In this sense, thanks to colorimetry, it was possible to know the colour changes of the mock-ups in the different stages of the experiment. For that, repetitive measurements of the central point of the studied area were carried out providing a relative standard deviation of less than 2%. The chroma (C*) and hue (h) values of the mock-ups at each stage of the experiment are shown in Figure 2 (left). At T0, the mock-ups presented a very similar colour, as can be seen in the figure with a very low dispersion of all the mock-up which indicated that the colour at the beginning was homogeneous. After the staining step (T1), the measurements showed also low dispersion at the values of C* and h (7% and 1% of relative standard deviation, respectively), revealing their homogeneous colour. After the first cleaning step (T2), as can be seen in the Table S1, the highest ΔE (T2-T1) values were obtained for all the gels containing citrate and EDTA (KJ2, KJ3, KD2, KD3, AG2, and AG3), which were the only ones that started to reveal the white colour of the marble. However, as can be seen in Figure 2 (left), the C* and h values were still far from the original mock-ups (T0). After the second step (T3), cleaning was improved in all cases, achieving values for C* and h that were very close to those of the original mock-ups. The gel that achieved the closest colour to the original surface of the mock-ups was konjac with EDTA (KJ3), konjac with citrate and kudzu with EDTA (KJ2 and KD3) following closely behind.

Figure 2 (right) shows two different colour variations. T3-T1 represents the cleaning of the copper patina, while T3-T0 represents the similarity between the original mock-up and after the cleaning. Therefore, the greater the colour variation in T3-T1, the greater the copper removal, and the smaller the colour variation in T3-T0, the more similar the cleaned mock-up will be to the original. As can be seen, after this second cleaning, the citrate and EDTA gels had succeeded in cleaning the surface of the mock-ups, achieving a great chromatic similarity to the marble before staining. At the end of the cleaning stages, the colour difference between the original and the cleaned mock-ups (T3-T0) was less than 5 for the konjac mock-ups with citrate and EDTA (KJ2 and KJ3), which means that the colour difference is not easily distinguishable, an important achievement for the cleaning.

**Figure 3:**
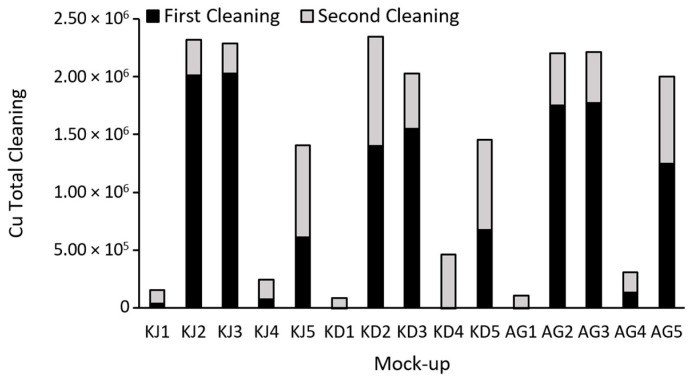
Difference in Cu counts between the stained mock-ups (T1) and after each cleaning. First cleaning (T2-T1) and second cleaning (T3-T2). The counts belong to the sum of the XRF spectra of the surface mappings of the mock-ups.

On the other hand, a semi-quantitative elemental comparison was also carried out by XRF mapping. Figure 3 shows the difference in Cu achieved in the first and second cleanings. The greater the difference, the greater the removal of Cu from the mock-ups’ surfaces, which translates to better efficiency. After both cleanings, generally the highest cleaning values were found after the application of the gels with citrate and EDTA. After the second application in the case of the citrate and EDTA gels, the extraction of the cooper was the 11% and 40% of the total copper extracted after both cleanings, being necessary this second cleaning to achieve good results. It is important to note that with gluconic acid, a greater extraction was achieved compared to the other gels (an average of 49% of the total cleaning) after the second cleaning which proved to be crucial. In fact, the agar with gluconic acid (AG5) gel even achieved a copper extraction comparable to that of kudzu with EDTA (KD3). However, despite of the AG5 gel, again, citrate and EDTA gels obtained the best results.

Comparing the colorimetric analyses with the XRF analyses, it can be seen that both citrate and EDTA gels were the best at removing Cu from the surface of the marble mock-ups. It should be noted that the two chelating agents were equally effective for all three types of gels. However, there are discrepancies between the two analytical methods for the gels with gluconic acid. The konjac with gluconic acid gel showed a low recovery of the original colour in the colorimetric analysis, while the XRF shows that the amount of Cu remaining on the surface of the mock-up is similar to that of the other two gels of gluconic acid. This may be due to the fact that although much of the Cu has been removed, there is still no apparent colour change. This highlights the importance of combining both techniques when assessing surface cleanliness.

#### 2.2.3. Gels Characterization

**Figure 4:**
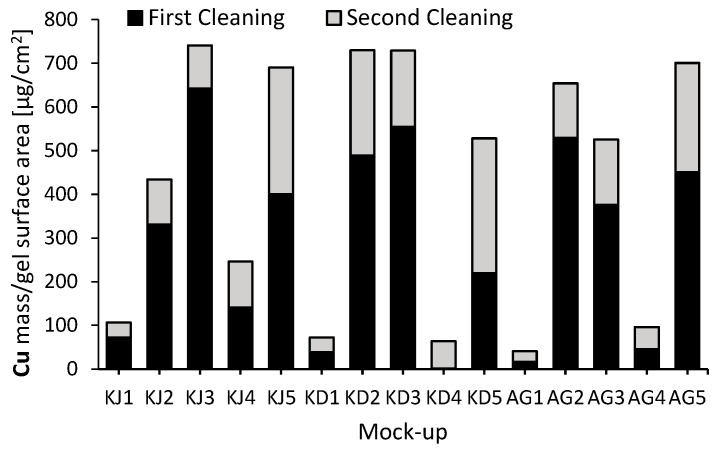

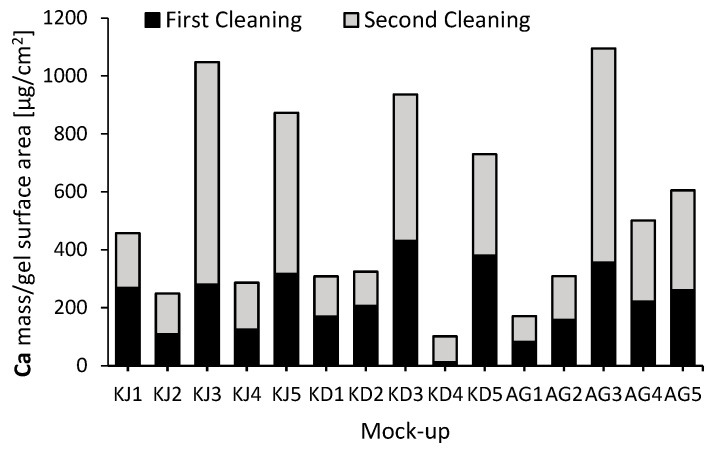
Copper and calcium extracted by each gel after its application.

The quantification of the material extracted by the gels was carried out by inductively coupled plasma mass spectrometry (ICP-MS). For that, the obtained results were adjusted to the mass of cooper extracted per area of gel (μg/cm^2^). The extraction of Cu shown in Figure 4 was higher during the first cleaning compared to the second, which is logical in cases where a large amount of copper had already been extracted after the first cleaning.

The gels without a chelating agent and with oxalate had the worst copper extraction results. However, among the non-chelating gels, konjac (KJ1) had the highest Cu extraction, with 48% more extraction than kudzu (KD1) and 163% more than agar (AG1), to which a certain intrinsic extraction capacity is attributed. Thus, the selected natural gels improve the extraction capacity of agar. In the case of gels doped with oxalate, higher Cu extraction was also achieved with konjac (KJ4), tripling the extraction values of kudzu and konjac (KD4 and AG4). Again, a higher extraction was achieved with the target natural gels, and in both cases konjac was the best.

The gels which extracted the highest amount of copper were konjac containing EDTA (KJ3) and gluconic acid (KJ5), kudzu containing citrate (KD2) and EDTA (KD3), and agar containing citrate (AG2) and gluconic acid (AG5). It is worth noting the less effectiveness of the konjac with citrate gel (KJ2), with 37% less extraction compared to kudzu and agar (KD2 and AG2). This was in contrast to the results observed with other analytical techniques, where the performance of the three citrate-doped gels did not vary as much.

EDTA with agar (AG3) was the reference gel and, as can be seen in Figure 4, its copper cleaning capacity was improved by combining EDTA with the other two gels. An improvement in extraction of 41% was obtained with konjac (KJ3) and 39% with kudzu (KD3).

**Figure 5:**
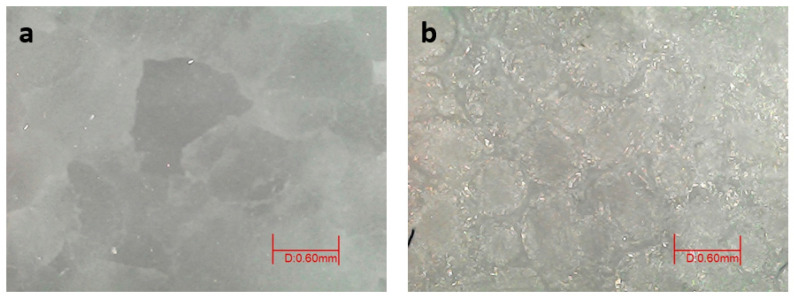
Surface of the marble mock-ups after application of the gels for cleaning the copper patina. On the surface treated with citrate gels no degradation is observed (**a**), whereas on the surface treated with EDTA gels, several pits are observed (**b**).

On the other hand, it was also interesting to observe the amount of calcium extracted in order to assess the damage caused to the calcite during the cleaning of its surface. The gels with the EDTA chelator (KJ3, KD3, and AG3) had the highest calcium extraction (Figure 4), followed by the gels with gluconic acid (KJ5, KD5, and AG5). This is due to the high affinity of these chelators for calcium. The greater extraction of calcium by these gels caused the degradation of the marble during cleaning, as can be seen in Figure 5. On the other hand, this degradation effect was not so evident after the application of the other gels.

All things considered, the best compositions could be suggested for each type of gel to achieve great copper extraction minimizing the damage of the marble surface. The gels with citrate had the best results when it came to extracting large amounts of copper without damaging the marble surface, achieving 83% with konjac and 139% with kudzu of the copper extraction achieved with the reference gel, agar with EDTA. The gels doped with gluconic acid also achieved a high extraction of copper, averaging 122% of that extracted by the reference gel. However, they extracted more than twice the amount of calcium from the marble surface in comparison to the citrate gels. 

### 2.3. Application after Drying

Some differences were observed in the application of the gels left to dry, but most results were similar. The consistency of the gels when dried was rigid and most of them had detached from the surface of the mock-ups, having lost all their adhesion power during the application time. As with the two 8-hour applications, the citrate- and EDTA-doped gels generally gave the best copper cleaning results, followed by the gluconic acid-doped gels. The most notable difference in this single application method was the effect observed with the konjac gels. These seemed to produce a strong adhesion to the copper patina when dried, so that excellent cleaning results were obtained even without the use of chelating agents, thanks to the maintained contact of the gel with the surface during the entire application time.

The results from the colorimetric measurements (Table S2) indicated that longer application times notably increase the obtained results. Thus, the colour variation calculated between the copper patina mock-ups and the cleaned mock-ups (T2-T1) was significantly higher than in the 8 h applications. The unique gels that showed practically no colour variation were the kudzu and agar gels without chelating agent. This contrasts with the results of the 8 h applications, where the gels showed some intrinsic capacity to clean the surface. It must be noted that in this particular application, the gels became dry, whereas in the other application, they were removed while still wet. It is evident that when the gels are wet, copper migrates from the marble to the gels. However, the opposite effect was observed during dryness, where copper is once again transferred back to the marble. Therefore, the longest application times are unsuitable for these gels if it involves them getting dry.

The XRF data (Table S3) indicated high extraction of konjac gels. There were no large variations in the effect of the different chelator on this gel, since the values for copper extraction were very similar, showing only an 11% variation. It is worth noting that the highest copper removal was achieved by citrate and gluconic acid (KJ2 and KJ5). So, all the chelating agents had similar extraction rates probably due to the long application time without loss of contact with the surface that, in this case, was positive. Extraction differences between the different chelators were discernible in the case of agar and kudzu gels. Citrate was the chelating agent that extracted the most copper in the agar case (AG2), improving the reference gel (AG3). In a similar manner, citrate demonstrated the highest extraction result with kudzu (KD2), extracting 41% more copper than the rest of the chelators.

Quantitative analysis conducted through ICP-MS (Table S4) supported the results already observed through XRF in relation to copper extraction. However, this analysis was useful to observe that the EDTA-doped gels extracted the most calcium in all cases. Agar with EDTA (AG3) gel achieved the highest calcium extraction, which was 25 times higher than the average extracted by gels doped with other chelators. Additionally, there was a 33% increase in the extraction of calcium when compared with the two 8 h applications. Therefore, longer application times are critical for the marble in the case of agar with EDTA. However, in the case of the other chelators and gels, this effect is not so evident despite of the increase in the application time.

## 3. Conclusions

During this experiment, it was possible to make a homogeneous and reproducible copper patina on marble mock-ups. In addition, gels of different compositions were prepared and applied to clean these mock-ups. Thanks to the reproducibility of the copper patina creation phase, it was possible to compare the cleaning efficiency of the different gels.

The gels analysed were prepared using agar, kudzu, and konjac which provide the gels with optimal and more uniform consistencies but with different percentages. The simplest gels to prepare were the agar gels because their viscosities during their preparation minimized the loss of material and facilitated the cleaning of the material used during its preparation. In contrast, konjac stands out because it does not require a heat source during preparation, allowing the possibility of in situ preparation and with no requirement of energy for its preparation in line with green chemistry. It is essential to highlight that the compatibility of kudzu with chelating agents has been demonstrated, having inherent antimicrobial properties.

The analytical techniques used showed that gels prepared with EDTA, citrate, and gluconic acid were the most effective in removing copper from the surface of the marble mock-ups after two 8-h applications. However, the ICP-MS analysis, accompanied by images taken with a digital magnifier, revealed the strong degradation associated with the use of the EDTA-doped gels. Although EDTA has the highest formation constant to form complexes with Cu(II), it is not recommended to use this chelating agent in the preparation of gels for marble cleaning. Therefore, the best results were obtained with kudzu with citrate or konjac with gluconic acid, both of which were more gentle on the surface than the reference gel of agar with EDTA.

Regarding application times, it has been demonstrated that the long application times drastically affect the extraction capacity of the gels without chelating agents. Moreover, the gels doped with EDTA extracted a significantly greater quantity of calcium. In addition, konjac seems to have intrinsic characteristic that improves the extraction with long application times. Therefore, this gel is the most appropriate for these conditions.

In summary, this study demonstrates the improved efficiency of the selected natural gels and the incompatibility of EDTA because it extracts a large amount of calcium resulting in the degradation of the marble. Moreover, studies indicate that EDTA acts as a contaminant by mobilising heavy metals [23]. This is why the European Union has issued a risk assessment document to regulate its usage, which must be taken into account when using EDTA in outdoor heritage structures [24]. The fact that the suggested natural gels doped with citrate or gluconic acid increase their efficacy—when compared to agar doped with EDTA—in addition to the information stated above, emphasises that these two chelators with konjac or kudzu gels are a legitimate alternative to EDTA for removing copper patinas on marble surfaces.

## 4. Materials and Methods

### 4.1. Preparation of Laboratory Mock-Ups

**Figure 6:**
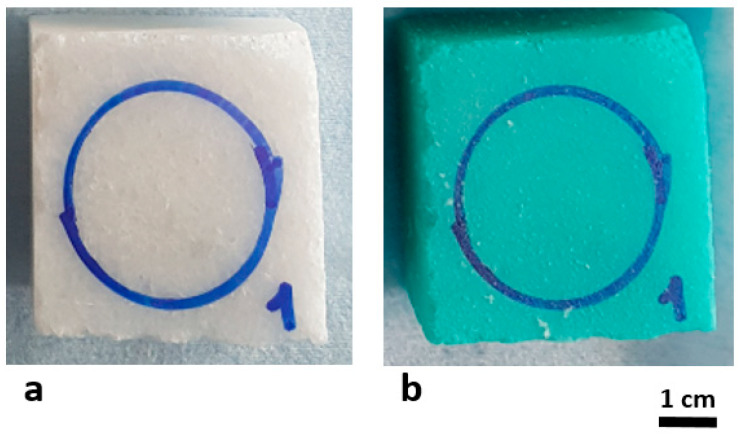
Image of the marble mock-ups before (**a**) and after staining (**b**).

The marble blocks used were “Blanco Ibiza”, supplied by the company ERCOR Stone (Bilbao, Spain). Fifteen marble mock-ups were cut to a size of 5 cm × 5 cm × 2 cm, to have individual pieces to apply the gels. To create the copper patina, a method widely used in the literature [8] was used. The method consisted of completely submerging the mock-ups in a 0.1 M solution of CuSO_4_·2H_2_O for 72 h and was chosen for its simplicity and the capacity to stain multiple mock-ups simultaneously. The mock-ups were then removed and dried at room temperature and they acquired a turquoise colour, which at first glance appeared to be homogeneous over the entire surface of the mock-ups (Figure 6).

### 4.2. Gels Preparation

Three different types of gel were used in this study. Agar (Fluka Analytical, Spain) gel was used as the reference gel as it is widely used in heritage cleaning. The other two gels used were kudzu (Mitoku Co., Tokyo, Japan) and konjac (Biotiva, Straßlach-Dingharting, Germany) as alternatives to agar.

In addition, four chelating agents were used in the preparation of the cleaning gels. These chelating agents were selected based on their stability constants to form complexes with the Cu^2+^ ion. Chelating agents with a complexation constant with Cu(II) similar to that with EDTA (Table 2) were chosen [25,26]. In addition to the constants, it was considered that the selected chelating agents were not toxic to the environment or living organisms, in line with the principles of green chemistry. Thus, the reference compound was sodium EDTA (C_10_H_14_N_2_Na_2_O_6_·2H_2_O, Panreac, Castellar del Vallès, Spain), while the others were sodium citrate (C_6_H_5_Na_3_O_7_·2H_2_O, Sigma-Aldrich, Saint Louis, MO, USA), sodium oxalate (C_2_Na_2_O_4_, Sigma-Aldrich), and gluconic acid (C_6_H_11_NaO_7_, Sigma-Aldrich).

The three types of gel were used without chelating agent to evaluate the intrinsic capacity to clean surfaces and with each of the chelating agents separately. Each type of gel had a different preparation method, but all were prepared using the same volume of ultrapure water (30 mL) and the same percentage by weight of chelating agent (3%) based on previous works for EDTA [3,9]. Due to the different properties of the gels, different percentages by weight were used for their preparation: 2% for agar, 12% for kudzu, and 6% for konjac. These percentages were selected to obtain a similar stiffness and stability for all gels, using agar gel as reference.

To prepare the agar gels, agar was first dissolved in water at room temperature and then heated to 85 °C. Once this temperature was reached, the contents were poured into a Petri dish and left to cool and jellify. Kudzu was also prepared by dissolving it in water at room temperature and then heating it to about 65 °C. It was kept at this temperature with constant stirring until the mixture began to take on a viscous consistency and the whitish colour turned yellowish. It was then poured into a Petri dish and left to cool. Finally, to prepare konjac gel, all that was needed was to mix water at room temperature with konjac and stir until it quickly reached a viscous consistency. Again, the contents were poured into a Petri dish. All gels were prepared at the same time and allowed to settle long enough to ensure that they were at room temperature and had reached the desired stiffness (at least one hour prior to their application). All the Petri dishes used had a diameter of 8.5 cm.

### 4.3. Gels Application

The application of the gels was made using a circular patch of 3 cm diameter that was cut out of each Petri dish and carefully placed in the centre of a mock-up. The perimeter of the application was marked to guarantee the area to be characterised before and after the application. A gel was applied to each mock-up, giving a total of 15 applications, 1 for each gel composition. The patch compositions and their coding are shown in Table 3. The patches were coded “KJ” for konjac, “KD” for kudzu, and “AG” for agar plus adding a number from 1 to 5 in the following order of composition: without chelator, with sodium citrate, with EDTA, with sodium oxalate, and with gluconic acid.

**Figure 7:**
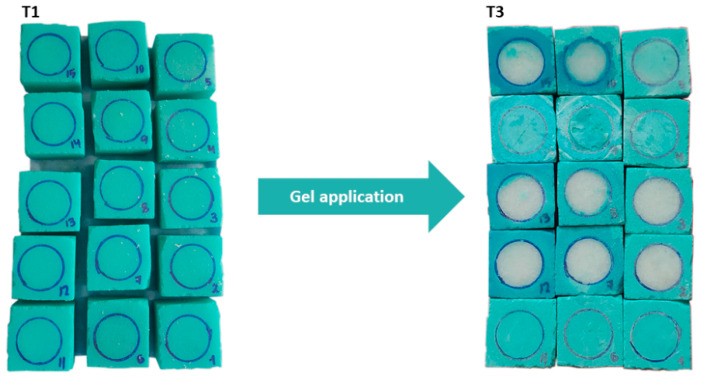
Mock-ups before (T1) and after gel application (T3).

After 8 h, the patches had a bluish tinge and were removed (Figure 7). An 8-h period was chosen because it represents the length of a typical working day. Finally, new gels were prepared following the same procedure for a second application to the same mock-ups.

The study was centred on the double application of 8 h, but other 15 new mock-ups were prepared to study the application of the gels also until they became dry. The time for the gels to dry can vary greatly outdoors, but in this case, in a closed environment such as a laboratory it was 3 days.

### 4.4. Marble Characterization

The marble mock-ups were analysed at each stage of the experiment. Four stages were defined for the monitoring of the cleaning process: before the formation of the copper patina (T0), after the formation of the copper patina (T1), after the first application of the gels (T2), and after the second application of the gels (T3).

The marble mock-ups and the copper patina created were characterized by Raman spectroscopy using a Renishaw inVia Raman spectrometer (Renishaw, Wotton-under-Edge, UK) coupled to a Leica DMLM microscope (Leica, Wetzlar, Germany) to characterise the minerals formed and ensure that they are the same as those found in real patinas. Measurements were made with a 532 nm laser at 5% laser power using a 20x objective.

Colorimetry was used for the visual examination of the surface colour using a PCE Instruments PCE-CSM 1 colorimeter with 4 mm measuring aperture to quantitatively assess the colour change caused by both cleaning and patina formation. Three measurements were taken for each mock-up and used to obtain the L*, a*, b*, C*, and h values of the CIELAB and CIECH spaces. In addition, the colour difference (ΔE*) was calculated from the L* a* b* values. The ΔE* value represents the distance between two points in a three-dimensional space, so the formula is [27]:

(1) $${\Delta E}^{*}=\sqrt{{\left({\Delta L}^{*}\right)}^{2}{+\left({\Delta L}^{*}\right)}^{2}+{\left({\Delta L}^{*}\right)}^{2}}$$

X-ray fluorescence imaging (XRF) was used for elemental analysis of the surface of the mock-ups at each stage of the experiment. In this way, the homogeneity of the patina generation was assessed by evaluating the copper deposited on the surface and the cleaning efficiency by monitoring the copper variation. Moreover, the suitability of the cleaning was evaluated monitoring the calcium variation before and after the cleaning process. XRF mapping was performed using an M4 Tornado ED-XRF spectrometer (Bruker Nano, Berlin, Germany) equipped with an Rh anode tube operating at up to 50 kV with a maximum current of 700 μA. The mock-ups were mapped with a spot size of 1150 μm and an acquisition time of 500 ms for each spot, with dimensions of 29.5 mm × 29.5 mm. The net counts of the copper K_α_ lines from the sum spectrum of the mapping were used to semiquantitatively assess the variation of copper over the surface of the mock-ups during the stages.

### 4.5. Gels Characterization

Inductively coupled plasma mass spectrometry (ICP-MS) was used to determine the amount of copper and calcium extracted from the gels after the cleaning (T3). The analysed elements were copper (Cu) and calcium (Ca). Copper was used to assess the extraction of patina, while calcium was used to measure the degradation of the marble surface. Prior to analysis, the gels were digested in a 5 mL 1:3 mixture of HCl (36%, Tracepur) and HNO_3_ (69%, Tracepur) and left in an ultrasonic bath for 30 min. The extracts obtained from the acid digestions were filtered using 20 mL syringes (B. Braun Omnifix) and 0.45 μm filters (Teknokroma, Sant Cugat del Vallés, Spain). Finally, 1:10 dilutions were made to adjust to the calibration curve and HNO_3_ was adjusted to 1%.

The ICP-MS analysis was performed using a Perkin Elmer NexION 300 with an OneNeb pneumatic nebuliser, cyclonic spray chamber, and nickel cones. The instrument was used with a power of 1600 W and integration times of 1000 ms divided into 20 sweeps of 50 ms each. Calibrations were performed with Alfa Aesar Specpure^®^ solutions using 9Be, 45Sc, 115In, and 209Bi as internal standards and He as collision gas (4 mL/min).

## Figures and Tables

**Table 1:** pH values of the different gel compositions.

Chelating Agent	Konjac	Kudzu	Agar
-	6.07	5	5.64
+ sodium citrate	7.31	7.31	7.75
+ EDTA	4.51	4.49	4.30
+ sodium oxalate	6.56	6.42	6.67
+ gluconic acid	6.37	6.30	6.30

**Table 2:** Formation constants (K_f_) for Cu(II) complexes with the different ligands studied [25,26].

Chelating Agents (Ligands)	Log K_f_
EDTA	18.70
Citrate	14.20
Oxalate	8.50
Gluconic acid	6.08

**Table 3:** Gels composition and coding.

Chelating Agent (3%)	Konjac (6%)	Kudzu (12%)	Agar (2%)
-	KJ1	KD1	AG1
+ sodium citrate	KJ2	KD2	AG2
+ EDTA	KJ3	KD3	AG3
+ sodium oxalate	KJ4	KD4	AG4
+ gluconic acid	KJ5	KD5	AG5

## Data Availability

The data presented in this study are openly available in the article.

## Supplementary Materials

The following supporting information can be downloaded at: https://www.mdpi.com/article/10.3390/gels9110843/s1, Table S1: Colour variation values ΔE of each of the cleanings; Table S2: Data from colorimetric analysis obtained during cleaning of the application after drying; Table S3: Data from XRF analysis obtained during cleaning of the application after drying. Difference in Cu counts between the stained mock-ups and after cleaning; Table S4: Data from ICP-MS analysis obtained during cleaning of the application after drying. Copper and calcium extracted by each gel after its application.
