# Supplementary material for: Looking for Novel Natural Gels to Improve Cleaning Methods for Bronze Leachates on Marble
Source: Gels. 2023 Oct 25;9(11):843. doi: 10.3390/gels9110843 (PMC10670686; doi:10.3390/gels9110843)
Supplement: Supplementary file 1 [file gels-09-00843-s001.zip › gels-2654111-supplementary.pdf]

Supplementary Materials

Table S1. Colour variation values ΔE of each of the cleanings. Coloured in green are values greater than 5, indicating an easily noticeable change in colour.

	First Cleaning	Second Cleaning	Total Cleaning
	ΔE (T2-T1)	ΔE (T3-T2)	ΔE (T3-T1)
KJ1	8	2	6
KJ2	16	6	22
KJ3	14	9	22
KJ4	4	5	6
KJ5	3	5	3
KD1	3	1	2
KD2	12	11	22
KD3	13	9	21
KD4	5	2	6
KD5	3	17	20
AG1	3	3	5
AG2	16	5	21
AG3	15	5	20
AG4	8	4	12
AG5	9	14	22

Table S2. Data from colorimetric analysis obtained during cleaning of the application after drying.

	T0			T1			T2			(T2-T1)
	L	a*	b*	L	a*	b*	L	a*	b*	ΔE
KJ1	71.51	−1.31	−2.7	54.4	−45.63	−15.2	64.99	−15.44	−9.47	32.50
KJ2	71.73	−1.4	−2.92	53.68	−45.27	−14.84	61.12	−2.27	−2.56	45.33
KJ3	71	−1.36	−2.82	53.38	−48.9	−14.86	63.22	−3.93	−3.44	47.43
KJ4	71.6	−1.3	−2.6	54.98	−36.59	−14.94	69.42	−3.94	−4.24	37.27
KJ5	72.43	−1.36	−2.73	54.05	−43.24	−15.09	56	−4.17	−3.47	40.81
KD1	70.36	−1.3	−2.42	54.14	−40.92	−15.39	54.76	−39.42	−15.3	1.63
KD2	73.33	−1.31	−2.63	55.05	−41.65	−14.82	64.04	−3.08	−2.71	41.41
KD3	71.04	−1.33	−2.64	55.5	−31.92	−14.67	64.36	−4.24	−3.85	31.01
KD4	69.08	−1.31	−2.76	54.44	−47.6	−15.65	68.92	−28.92	−17.01	23.67
KD5	70.65	−1.35	−2.88	54.2	−39.33	−14.99	54.08	−14.5	−7.48	25.94
AG1	70.75	−1.32	−2.75	54.54	−40.08	−14.97	54.73	−39.86	−14.71	0.39
AG2	71.44	−1.31	−2.72	53.34	−52.4	−14.78	57.42	−2.8	−2.73	51.21
AG3	71.31	−1.27	−2.52	54.29	−39.26	−14.92	56.89	−11.49	−6.53	29.13
AG4	72.28	−1.28	−2.64	53.85	−46.32	−14.53	69.82	−22.1	−14.59	29.01
AG5	76.68	−1.01	−2.12	53.33	−44.11	−14.75	49.93	−26.07	−13.63	18.39

Table S3. Data from XRF analysis obtained during cleaning of the application after drying. Difference in Cu counts between the stained mock-ups and after cleaning. ND: no difference.

	Difference in Cu Counts
KJ1	2,890,264
KJ2	4,083,784
KJ3	4,718,721
KJ4	3,673,585
KJ5	4,535,227
KD1	ND
KD2	3,786,088
KD3	2,151,944
KD4	2,376,877
KD5	2,146,642
AG1	ND
AG2	4,625,610
AG3	3,984,632
AG4	1,141,770
AG5	2,081,537

Table S4. Data from ICP-MS analysis obtained during cleaning of the application after drying. Difference in Cu counts between the stained mock-ups and after cleaning. Copper and calcium extracted by each gel after its application.

	Cu (μg/cm^2)	Ca (μg/cm^2)
KJ1	1813	48
KJ2	2027	29
KJ3	2609	448
KJ4	2157	78
KJ5	2512	73
KD1	70	13
KD2	2566	49
KD3	1036	689
KD4	488	25
KD5	1151	125
AG1	106	47
AG2	2348	53
AG3	2179	1459
AG4	666	77
AG5	1274	77
